# Low serum eicosapentaenoic acid / arachidonic acid ratio in male subjects with visceral obesity

**DOI:** 10.1186/1743-7075-10-25

**Published:** 2013-03-12

**Authors:** Kana Inoue, Ken Kishida, Ayumu Hirata, Tohru Funahashi, Iichiro Shimomura

**Affiliations:** 1 Department of Metabolic Medicine, Graduate School of Medicine, Osaka University, Suita, Osaka 565-0871, Japan; 2 Kishida Clinic, 5-6-3, Honmachi, Toyonaka, Osaka 560-0021, Japan; 3 Department of Metabolism and Atherosclerosis, Graduate School of Medicine, Osaka University, Suita, Osaka 565-0871, Japan

**Keywords:** Arachidonic acid, Eicosapentaenoic acid, Docosahexaenoic acid, Visceral fat, Metabolic syndrome, Obesity

## Abstract

**Background:**

Visceral fat accumulation is caused by over-nutrition and physical inactivity. Excess accumulation of visceral fat associates with atherosclerosis. Polyunsaturated fatty acids have an important role in human nutrition, but imbalance of dietary long-chain polyunsaturated fatty acids, especially low eicosapentaenoic acid (EPA) / arachidonic acid (AA) ratio, is associated with increased risk of cardiovascular disease. The present study investigated the correlation between EPA, docosahexaenoic acid (DHA), AA parameters and clinical features in male subjects.

**Findings:**

The study subjects were 134 Japanese with diabetes, hypertension and/or dyslipidemia who underwent measurement of visceral fat area (eVFA) by the bioelectrical impedance method and serum levels of EPA, DHA and AA. EPA/AA ratio correlated positively with age, and negatively with waist circumference and eVFA. Stepwise regression analysis demonstrated that age and eVFA correlated significantly and independently with serum EPA/AA ratio. Serum EPA/AA ratio, but not serum DHA/AA and (EPA+DHA)/AA ratios, was significantly lower in subjects with eVFA ≥100 cm^2^, compared to those with eVFA <100 cm^2^ (p=0.049). Subjects with eVFA ≥100 cm^2^ were significantly more likely to have the metabolic syndrome and history of cardiovascular diseases, compared to those with eVFA <100 cm^2^ (p<0.001, p=0.028, respectively).

**Conclusions:**

Imbalance of dietary long-chain polyunsaturated fatty acids (low serum EPA/AA ratio) correlated with visceral fat accumulation in male subjects.

**Clinical trial registration number:**

UMIN000002271

## Background

Obesity, especially excess accumulation of visceral fat, is upstream of obesity-related disorders including atherosclerotic cardiovascular disease [1]. Visceral fat accumulation represents positive energy balance, conditioned by genetic and environmental factors, such as high-fat and/or high sucrose diet, physical inactivity, sleep-disorders, and mental stress [1]. Polyunsaturated fatty acids have an important role in human diet, both in the prevention and treatment of pathologies [2,3]. Previous studies reported that an imbalance of dietary long-chain polyunsaturated fatty acids, especially low omega-3/omega-6 polyunsaturated fatty acids ratio, is associated with increased risk of cardiovascular disease [4-6]. To our knowledge, the relationship between visceral fat volume and omega-3/omega-6 polyunsaturated fatty acid ratio has not yet been characterized. The present study investigated the correlation between omega-3 polyunsaturated fatty acid (eicosapentaenoic acid [EPA], docosahexaenoic acid [DHA]), omega-6 polyunsaturated fatty acid (arachidonic acid [AA], and clinical features in male subjects.

## Methods and procedures

### Study population

The study subjects were 134 consecutive Japanese subjects with diabetes, hypertension and/or dyslipidemia, who visited the outpatient clinic of “Diabetes & Metabolic Station” at Osaka University Hospital, between September 2009 and August 2012. The Medical Ethics Committee of Osaka University approved this study. All participants were Japanese and each gave a written informed consent. This study (ADMIT study) is registered under number UMIN000002271.

### Definition of coronary artery diseases

Documented coronary artery diseases (CAD) encompassed one or more of the following: history of stable with documented CAD evaluated with stress electrocardiography, such as ergometry or double master’s two-step test, multi-detector row computed tomography or coronary angiography; history of previous acute coronary syndrome including unstable angina and myocardial infarction evaluated with coronary angiography and underwent percutaneous coronary intervention or coronary artery bypass graft surgery.

### Anthropometric data and laboratory tests

Anthropometric variables [height, weight and waist circumference (WC)] were measured in the standing position. WC at the umbilical level was measured with a non-stretchable tape in late expiration while standing (in cm). Body mass index (BMI) was calculated using the formula [*weight*(*kg*)/*height*(*m*)^2^]. Visceral fat area was estimated by the bioelectrical impedance analysis method (eVFA), as reported previously by our group [7]. Briefly, the voltage recorded at the flank to the flow of current between the umbilicus and the back correlates significantly with visceral fat area and is not influenced by the amount of subcutaneous fat. We demonstrated previously that eVFA correlates significantly with that determined by computed tomography [7]; the coefficient of variation of the bioelectrical impedance analysis method with the computed tomography value was 0.89% in the standing posture and late expiration.

Blood pressure was measured with a standard mercury sphygmomanometer on the right arm in the supine position after at least 10-minute rest. After overnight fasting state, venous blood samples were collected for measurements of blood glucose, hemoglobin A1c (HbA1c) [National Glycohemoglobin Standardization Program (NGSP)], triglyceride, and high-density lipoprotein-cholesterol (HDL-C), uric acid, and creatinine. Low-density lipoprotein-cholesterol (LDL-C) was calculated with the Friedewald equation. Urine albumin-creatinine ratio (UACR) was calculated from a single spot urine specimen collected between morning and afternoon.Urine albumin concentration was determined by the immunoturbidimetric method (N-assay TIA MicroAlb, Nittobo Medical, Japan). Estimated GFR (eGFR) was calculated using the simplified Modification of Diet in Renal Disease equation modified by the appropriate coefficient for Japanese populations by gender, as described previously [8]. The homeostasis model assessment of insulin resistance (HOMA-IR) was calculated using the following formula: *HOMA* − *IR* (*milliunits per liter* × *milligrams per deciliter*) = [(*fasting IRI*(*microunit*/*mL*) × *fasting glucose* [*mg*/*mL*])/405]. Measurement of serum EPA, DHA and AA levels was outsourced to SRL (Tokyo, Japan). In brief, plasma lipids were extracted by Folch’s procedure, and then fatty acids (tricosanoicacid, C23:0, as the internal standard) were methylated with boron trifluoride and methanol. The methylated fatty acids were then analyzed using a capillary gas chromatograph (GC-2010; Shimadzu, Kyoto, Japan).

Subjects were divided according to smoking habit into current or ex-smokers and non-smokers. Diabetes mellitus was defined according to World Health Organization criteria or regular treatment with anti-diabetic agents. Dyslipidemia was defined as total cholesterol concentration of ≥220 mg/dL, triglyceride concentration ≥150 mg/dL, HDL-C concentration <40 mg/dL, or regular treatment with anti-lipidemic agents. Hypertension was defined as systolic blood pressure ≥140 mmHg, diastolic blood pressure ≥90 mmHg, or regular treatment with anti-hypertensive agents. Patients with a previous diagnosis of dyslipidemia, hypertension, or diabetes mellitus and receiving drugs for any of these conditions were also included in this study. The metabolic syndrome was diagnosed according to the Japanese criteria for the metabolic syndrome [9].

### Statistical analysis

All values were expressed as mean±SD. Data of eVFA, and triglyceride levels showed skewed distribution, and were therefore log-transformed before analysis. Relationships between two continuous variables were analyzed using scatter plots and Pearson’s correlation coefficients. Data of the two groups were compared by the Student’s *t*-test or the Mann–Whitney test. The frequencies were compared between two groups by the χ2 test. The correlations between EPA,DHA, AA, EPA/AA, DHA/AA, (EPA+DHA)/AA and other parameters were first analyzed by simple regression analysis and then by multivariate stepwise analysis. In all cases, *p* values <0.05 were considered statistically significant. All analyses were performed with the JMP Statistical Discovery Software 8.0 (SAS Institute, Cary, NC).

## Results

### Subjects’ characteristics

**Figure 1:**
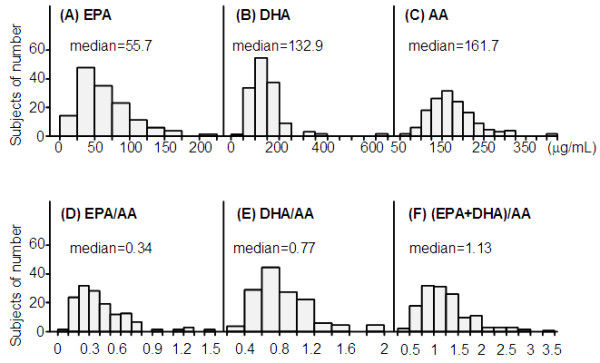
Distribution of eicosapentaenoic acid (EPA), docosahexaenoic acid (DHA), arachidonic acid (AA), EPA/AA ratio, DHA/AA ratio, (EPA+DHA)/AA ratio.

Table 1 summarizes the characteristics of all male subjects enrolled in this study. Visceral fat accumulation (eVFA ≥100 cm^2^) based on Japanese criteria of visceral fat accumulation [10] was identified in 76.9% (n=103/134). Figure 1 showed the distribution of EPA, DHA, AA, EPA/AA, DHA/AA and (EPA+DHA)AA ratios.

**Table 1:** Baseline characteristics

n (Male)	134
Age, years	64±12 (25–86)
Body mass index (BMI), kg/m^2^	25.6±3.9 (18.1-38.5)
Waist circumference (WC), cm	90.6±10.7 (68–130)
Estimated visceral fat area (eVFA), cm^2^	142±60 (36–371)
History of smoking (%)	82.1
Brinkman index	747±666 (0–3520)
Hypertension (%)	66.4
(calcium channel antagonist / angiotensin converting enzyme inhibitor or angiotensin receptor blocker / eplerenone / aliskiren fumarate / beta blockade / diuretics / alpha blockade )	(n=49/60/1/1/13/15/2)
Systolic blood pressure (SBP), mmHg	132±16 (94–180)
Diastolic blood pressure (DBP), mmHg	76±11 (52–111)
Diabetes (%)	78.4
(sulfonyl ureas / glinides / biguanides / pioglitazone /alpha glucosidase inhibitors / dipeptidyl peptidase-IV inhibitors / glucagon-like peptide-1 agonists / Insulin)	(n=42/3/25/24/21/11/1/20)
Fasting blood glucose (FBG), mg/dL	128±35 (56–261)
Hemoglobin A1c (HbA1c), (NGSP),%	6.9±1.1 (4.9-10.7)
HOMA-IR, units	3.1±2.4 (0.47-14.1)
Estimated glomerular filtration rate (eGFR), mL/min	69.5±17.7 (24.4-110.6)
Urine albumin-creatinine ratio (UACR), mg/g creatinine	175.1±552.0 (0.5-3958)
Dyslipidemia (%)	67.9
(statins / fibrates / ezetimibe / cholestimide / probucol)	(n=59/3/5/1/2)
Total cholesterol (T-cho), mg/dL	192±37 (112–335)
Low-density lipoprotein-cholesterol (LDL-C), mg/dL	112±33 (55–259)
Triglyceride (TG), mg/dL	142±91 (32–681)
High-density lipoprotein-cholesterol (HDL-C), mg/dL	53±16 (23–157)
Metabolic syndrome (%)	55.2
Eicosapentaenoic acid (EPA), μg/mL	63.8±35.7 (11.1-212.4)
Docosahexaenoic acid (DHA) , μg/mL	140.0±67.3 (41–610.3)
Arachidonic acid (AA), μg/mL	172.1±53.5 (72.5-413.4)
EPA/AA ratio	0.39±0.24 (0.09-1.41)
DHA/AA ratio	0.83±0.32 (0.35-1.98)
(EPA+DHA)/AA ratio	1.23±0.52 (0.45-3.27)
History of coronary artery diseases (CAD) (%)	39.6

Data are mean ± SEM (range), or number of subjects. HOMA-IR, homeostasis model assessment of insulin resistance.

### Correlation between serum levels of EPA, DHA, AA, EPA/AA DHA/AA, (EPA+DHA)AA ratios and clinical features

Table 2 and 3 summarize the results of correlation analyses of serum levels of EPA, DHA, AA, EPA/AA, DHA/AA, (EPA+DHA)/AA ratios with various clinical parameters. EPA correlated significantly and positively with LDL-C, HDL-C, DHA, and AA. Stepwise regression analysis that included LDL-C, HDL-C, DHA, and AA identified HDL-C, DHA, and AA correlated significantly and independently with EPA. DHA correlated significantly and positively with LDL-C, log-TG, EPA, and AA. Stepwise regression analysis that included LDL-C, log-TG, EPA, and AA identified log-TG, EPA, and AA correlated significantly and independently with DHA. AA correlated significantly and negatively with age, and positively with BMI, WC, eVFA, HOMA-IR, eGFR, LDL-C, log-TG, EPA, and DHA. Stepwise regression analysis that included age, BMI, eVFA, HOMA-IR, eGFR, LDL-C, log-TG, EPA, and DHA, demonstrated that age and DHA correlated significantly and independently with AA.

**Table 2:** Correlations between serum EPA, DHA, AA, and various parameters

	**Serum EPA**			**Serum DHA**			**Serum AA**		
	**Univariate**		**Multivariate**	**Univariate**		**Multivariate**	**Univariate**		**Multivariate**
	**r**	**p value**	**p value**	**r**	**p value**	**p value**	**r**	**p value**	**p value**
Age	0.066	0.449		−0.019	0.829		−0.381	<0.001	0.002
BMI	−0.049	0.577		0.064	0.465		0.216	0.012	0.952
WC	−0.083	0.342		0.042	0.632		0.172	0.048	-
Log-eVFA	−0.108	0.218		0.058	0.506		0.203	0.020	0.242
Brinkman index	−0.111	0.207		−0.139	0.114		−0.091	0.300	
SBP	0.049	0.571		0.101	0.244		−0.029	0.739	
DBP	−0.016	0.857		0.058	0.503		0.022	0.801	
FBG	−0.029	0.745		−0.013	0.886		0.024	0.786	
HbA1c	−0.059	0.510		−0.072	0.416		−0.059	0.503	
HOMA-IR	0.063	0.547		0.136	0.193		0.228	0.028	0.909
eGFR	0.045	0.608		−0.030	0.732		0.160	0.038	0.353
UACR	0.155	0.126		0.150	0.138		0.076	0.455	
LDL-C	0.233	0.008	0.142	0.256	0.004	0.424	0.236	0.007	0.835
HDL-C	0.181	0.037	0.030	0.113	0.196		0.122	0.161	
Log-TG	0.087	0.320		0.371	<0.001	<0.001	0.403	<0.001	0.390
EPA				0.757	<0.001	<0.001	0.251	0.004	0.259
DHA	0.757	<0.001	<0.001				0.517	<0.001	<0.001
AA	0.251	0.004	0.001	0.517	<0.001	<0.001			

Data are mean ± SEM (range), or number of subjects. Abbreviations as in Table 1. Parameters with p<0.05 in univariate analysis were subsequently entered into stepwise regression analysis as significant and independent variables. EPA (adopted parameters; LDL-C, HDL-C, DHA, AA), DHA (adopted parameters; LDL-C, Log-TG, EPA, AA), AA (adopted parameters; age, BMI, Log-eVFA, HOMA-IR, eGFR, LDL-C, log-TG, EPA, DHA).

**Table 3:** Correlations between serum EPA/AA, DHA/AA, (EPA+DHA)/AA ratios and various parameters

	**Serum EPA/AA ratio**			**Serum DHA/AA ratio**		**Serum (EPA+DHA)/AA ratio**	
	**Univariate**		**Multivariate**	**Univariate**		**Univariate**	
	**r**	**p value**	**p value**	**r**	**p value**	**r**	**p value**
Age	0.242	0.005	0.003	0.296	0.001	0.295	<0.001
BMI	−0.162	0.061		−0.127	0.144	−0.154	0.076
WC	−0.176	0.042	-	−0.117	0.179	−0.153	0.077
Log-eVFA	−0.212	0.015	0.026	−0.097	0.269	−0.157	0.071
Brinkman index	−0.081	0.696		−0.081	0.359	−0.066	0.453
SBP	0.079	0.679		0.135	0.120	0.100	0.251
DBP	−0.076	0.631		0.056	0.521	0.015	0.867
FBG	−0.009	0.919		−0.016	0.856	−0.016	0.851
HbA1c	−0.010	0.915		−0.015	0.863	−0.013	0.879
HOMA-IR	−0.064	0.543		−0.032	0.763	−0.047	0.654
eGFR	0.045	0.614		−0.079	0.373	−0.029	0.740
UACR	0.043	0.674		0.035	0.729	0.041	0.684
LDL-C	0.063	0.481		0.058	0.514	0.065	0.463
HDL-C	0.098	0.264		0.45	0.611	0.072	0.409
Log-TG	−0.154	0.076		0.074	0.396	−0.026	0.767

Data are mean ± SEM (range), or number of subjects. Abbreviations as in Table 1. Parameters with p<0.05 in univariate analysis were subsequently entered into stepwise regression analysis as significant and independent variables. EPA/AA (adopted parameters; age, eVFA).

The EPA/AA ratio correlated significantly and positively with age, and negatively WC and eVFA. Stepwise regression analysis demonstrated that age and eVFA correlated significantly and independently with EPA/AA ratio. The DHA/AA ratios and (EPA+DHA)/AA ratios correlated significantly and positively with age only.

**Figure 2:**
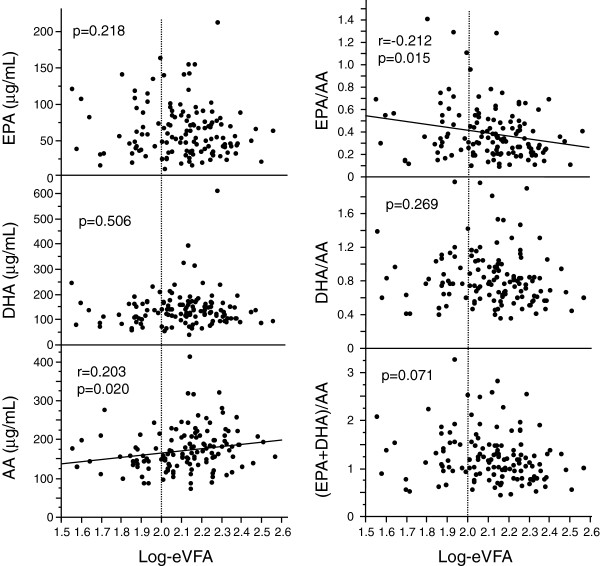
**Correlations between EPA, DHA, AA, EPA/AA ratio, DHA/AA ratio, (EPA+DHA)/AA ratio, and estimated visceral fat area (eVFA).** Pearson’s correlation coefficient was used to examine the relationship between EPA, DHA, AA, EPA/AA ratio, DHA/AA ratio, (EPA+DHA)+AA ratios, and eVFA. Abbreviations as in Figure 1.

Serum AA levels correlated positively with eVFA and serum EPA/AA and (EPA+DHA)/AA levels correlated negatively with eVFA, however, there was no significant correlation between EPA, DHA, DHA/AA ratio, and eVFA (Figure 2).

### Comparisons of clinical features in subjects with eVFA <100 cm^2^ and ≥100 cm^2^

**Figure 3:**
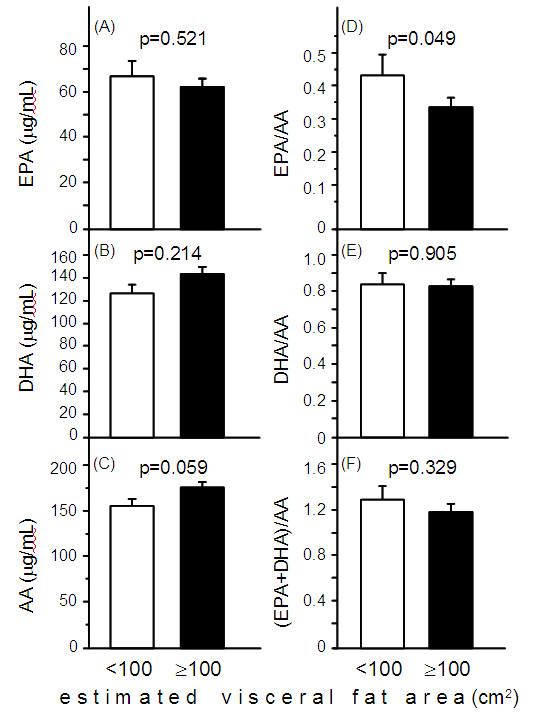
**Circulating levels of EPA, DHA, AA, EPA/AA ratio, DHA/AA ratio, and (EPA+DHA)/AA ratio in subjects with eVFA <100 cm**^**2 **^**and ≥100cm**^**2**^**.** Data are mean±SEM. Statistical comparisons by the Mann–Whitney U-test or Student’s *t*-test. Abbreviations as in Figure 1.

**Figure 4:**
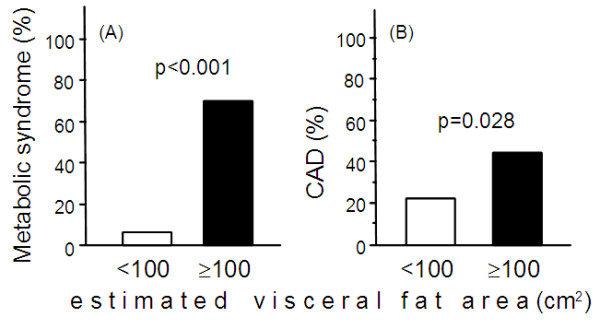
**Prevalence of metabolic syndrome and cardiovascular diseases (CAD) in subjects with eVFA <100 cm**^**2 **^**and ≥100cm**^**2**^**.** Data are mean±SEM. Statistical comparisons by the Mann–Whitney U-test or Student’s *t*-test. Abbreviations as in Figure 1.

Table 4 shows the characteristics of subjects with eVFA <100 cm^2^ and ≥100 cm^2^ based on Japanese criteria of visceral fat accumulation [9]. The proportion of patients with hypertension, and the levels of HOMA-IR and serum triglyceride were significantly higher in the eVFA ≥100 cm^2^ group, compared to the eVFA <100 cm^2^ group. Serum HDL-C levels were significantly lower in subjects with eVFA ≥100 cm^2^, than those with eVFA <100 cm^2^. Differences in serum EPA, DHA, and AA levels were not significant between the two groups (Figure 3A-C). Serum EPA/AA ratio, but not serum DHA/AA and (EPA+DHA)/AA ratios, was significantly lower in the eVFA ≥100 cm^2^ group, compared to the eVFA <100 cm^2^ group (Figure 3D-F). A larger proportion of subjects with eVFA ≥100 cm^2^ were diagnosed with the metabolic syndrome and cardiovascular diseases, compared to those with eVFA <100 cm^2^ (Figure 4A,B).

**Table 4:** **Comparison of various parameters between subjects with eVFA <100 cm**^**2 **^**and eVFA ≥100 cm**^**2**^

	**eVFA <100 cm**^**2 **^**group**	**eVFA ≥100 cm**^**2 **^**group**	**p value**
n (Male)	31	103	
Age, years	62±16 (25–86)	65±11 (27–85)	0.239
BMI, kg/m^2^	21.7±1.9 (18.1-25.8)	26.7±3.6 (20.6-38.5)	<0.001
WC, cm	78.0±4.6 (68–90)	94.4±9.0 (78–130)	<0.001
History of smoking (%)	67.7	86.4	0.045
Brinkman index	591±699 (0–3000)	795±652 (0–3520)	0.136
Hypertension (%)	41.9	73.8	0.001
SBP, mmHg	127±17 (94–168)	133±15 (98–180)	0.048
DBP, mmHg	71±9 (52–91)	78±11 (57–111)	0.005
Diabetes (%)	71.0	80.6	0.254
FBG, mg/dL	119±35 (80–216)	130±35 (56–261)	0.123
HbA1c, (NGSP),%	6.8±1.1 (4.9-8.9)	7.0±1.1 (5.4-10.7)	0.292
HOMA-IR, units	2.0±1.9 (0.47-8.0)	3.3±2.4 (0.63-14.1)	0.038
eGFR, mL/min	72.3±16.8 (43.0-103.4)	68.7±18.0 (24.4-110.6)	0.346
UACR, mg/g creatinine	244.6±869.9 (0.5-3958)	156.5±435.4 (1.0-2880)	0.519
Dyslipidemia (%)	54.8	71.8	0.075
T-cho, mg/dL	198±38 (137.8-271)	191±36 (112–335)	0.349
LDL-C, mg/dL	117±35 (68–182)	111±33 (55–259)	0.337
TG, mg/dL	87±35 (32–182)	159±97 (51–681)	<0.001
HDL-C, mg/dL	63±24 (31–157)	50±12 (56–261)	<0.001

Data are mean ± SEM (range), or number of subjects. Abbreviations as in Table 1. P values <0.05 were considered statistically significant.

## Discussion

The following were the major findings of the present study: 1) stepwise analysis demonstrated that age and eVFA correlated significantly and independently with serum EPA/AA ratio, and 2) Subjects with eVFA ≥100 cm^2^ had lower serum EPA/AA ratio and were more likely to have metabolic syndrome and history of CAD, compared to those with eVFA <100 cm^2^.

The preset subjects were males only. Another preliminary studies analyzed the difference of serum EPA/AA ratio in female subjects with eVFA <100 cm^2^ and ≥100 cm^2^ (n=100, data not shown). There was no significant difference of serum EPA/AA ratio in between female subjects with eVFA <100 cm^2^ and ≥100 cm^2^ (n=61/39, 0.37±0.27 versus 0.38±0.25, p=0.9236), and the two groups had lower serum EPA/AA levels. These data suggested that Japanese females may have an aversion to fish intake. Further studies including larger number of female subjects with eVFA ≥100 cm^2^ are needed to clarify the correlation between serum EPA/AA and eVFA.

In Japan, the percentage of high-fat food intake from animal sources per total daily calories has been increasing every year, based on the annual reports of the Ministry of Health, Labour and Welfare (http://www0.nih.go.jp/eiken/chosa/kokumin_eiyou/). The optimal dietary fat profile includes low intake of both saturated and omega-6 fatty acids and a moderate intake of omega-3 fatty acids [11]. Omega-3 and omega-6 fatty acids are essential because they are not synthesized by the body and must be obtained through diet or supplementation [12]. Omega-6 fatty acids, which are present in most seeds, vegetable oils, and meat, are prothrombotic and proinflammatory [12]. In contrast, omega-3 fatty acids, eicosapentaenoic acid and docosahexaenoic acid, which are present in most fish, such as salmon and tuna, and fish oil, are anti-thrombotic and anti-inflammatory [12]. The present study demonstrated a negative correlation between EPA/AA ratio and visceral fat area (Figure 2 and Table 3). Experimental and clinical studies suggest that insulin resistance could explain this negative relationship. Previous studies in diabetic rats demonstrated that long-term intake of EPA prevented insulin resistance, at least in part by improving hypertriacylglycerolemia [13]. Furthermore, low EPA/AA ratio, which is associated with insulin resistance, was also reported in young Japanese adults [14]. Another study demonstrated that the omega-3/omega-6 ratio correlated with HOMA-IR levels in subjects with the metabolic syndrome [15].

Our results demonstrated that subjects with eVFA ≥100 cm^2^ had lower serum EPA/AA ratio, but not serum DHA/AA, and were more likely to have history of CAD, compared to those with eVFA <100 cm^2^. A low ratio of n-3 to n-6 polyunsaturated fatty acids is associated with cardiovascular events [4,6,16-18]. However, either study had analyzed the patients with CAD or ischemic stroke, i.e. secondary preventive groups. The present study in lifestyle-related subjects suggests that low EPA/AA ratio may be important in primary prevention of cardiovascular events. Changes in food culture, such as low fish intake and high meat intake, i.e., low EPA/AA ratio, may explain at least in part the worldwide trend of increased prevalence of the metabolic syndrome and atherosclerotic cardiovascular diseases. Intervention with fish oil supplements high in EPA has shown a trend in reduced mortality in CAD patients with substantial reduction in the risk of sudden cardiac death [19,20]. Further intervention trials are needed to address the effect of balanced EPA/AA diet on the incidence of the metabolic syndrome.

In conclusion, the present study showed that low EPA/AA ratio correlated with visceral fat accumulation in male subjects, and subjects with eVFA ≥100 cm^2^ had lower serum EPA/AA ratio and were more likely to have the metabolic syndrome and history of cardiovascular disease, compared to those with eVFA <100 cm^2^. The balance of EPA/AA by lifestyle modification and medication (such as EPA-based medications) could be useful in reducing the prevalence of the metabolic syndrome and atherosclerosis.

### Study limitations

The present study has certain limitations. First, this is a cross-sectional study, making it difficult to establish a cause-effect relationship. Second, the results may not be applicable to females or non-Japanese populations. Third, there is bias in single center trials. Fourth, the number of patients may be relatively small. Finally, our study measured abdominal visceral fat area by the bioelectrical impedance method. Measurements of both visceral and subcutaneous fat areas by computed tomography are more accurate.

## Abbreviations

AA: Arachidonic acid; BMI: Body mass index; CAD: Coronary artery disease; DHA: Docosahexaenoic acid; EPA: Eicosapentaenoic acid; eVFA: Estimated visceral fat area; HbA1c: Hemoglobin A1c; HDL-C: High-density lipoprotein-cholesterol; LDL-C: Low-density lipoprotein-cholesterol; TG: Triglyceride; UACR: Urine albumin-creatinine ratio; WC: Waist circumference.

## Competing interests

TF is a member of the “Department of Metabolism and Atherosclerosis”, a sponsored course endowed by Kowa Co. Ltd. and a company researcher is dispatched to the course. All other authors declare no competing interests.

## Authors’ contributions

KI, KK and AH conceived of the study, participated in its design and coordination, extracted and analyzed data, and wrote the manuscript. KK also interpreted of data and reviewed/edited the manuscript. TF and IS contributed to the discussion and wrote the manuscript. All authors read and approved the final version of the manuscript.
